# Construction of a Public CHO Cell Line Transcript Database Using Versatile Bioinformatics Analysis Pipelines

**DOI:** 10.1371/journal.pone.0085568

**Published:** 2014-01-10

**Authors:** Oliver Rupp, Jennifer Becker, Karina Brinkrolf, Christina Timmermann, Nicole Borth, Alfred Pühler, Thomas Noll, Alexander Goesmann

**Affiliations:** 1 Center for Biotechnology, Bielefeld University, Bielefeld, Germany; 2 Cell Culture Technology, Bielefeld University, Bielefeld, Germany; 3 Department for Biotechnology, Universität für Bodenkultur Wien, Vienna, Austria; 4 ACIB, Austrian Center of Industrial Biotechnology, Graz and Vienna, Austria; 5 Bioinformatics and Systems Biology, Justus-Liebig-University, Giessen, Germany; CNRS UMR7622 & University Paris 6 Pierre-et-Marie-Curie, France

## Abstract

Chinese hamster ovary (CHO) cell lines represent the most commonly used mammalian expression system for the production of therapeutic proteins. In this context, detailed knowledge of the CHO cell transcriptome might help to improve biotechnological processes conducted by specific cell lines. Nevertheless, very few assembled cDNA sequences of CHO cells were publicly released until recently, which puts a severe limitation on biotechnological research. Two extended annotation systems and web-based tools, one for browsing eukaryotic genomes (GenDBE) and one for viewing eukaryotic transcriptomes (SAMS), were established as the first step towards a publicly usable CHO cell genome/transcriptome analysis platform. This is complemented by the development of a new strategy to assemble the ca. 100 million reads, sequenced from a broad range of diverse transcripts, to a high quality CHO cell transcript set. The cDNA libraries were constructed from different CHO cell lines grown under various culture conditions and sequenced using Roche/454 and Illumina sequencing technologies in addition to sequencing reads from a previous study. Two pipelines to extend and improve the CHO cell line transcripts were established. First, *de novo* assemblies were carried out with the Trinity and Oases assemblers, using varying k-mer sizes. The resulting contigs were screened for potential CDS using ESTScan. Redundant contigs were filtered out using cd-hit-est. The remaining CDS contigs were re-assembled with CAP3. Second, a reference-based assembly with the TopHat/Cufflinks pipeline was performed, using the recently published draft genome sequence of CHO-K1 as reference. Additionally, the *de novo* contigs were mapped to the reference genome using GMAP and merged with the Cufflinks assembly using the cuffmerge software. With this approach 28,874 transcripts located on 16,492 gene loci could be assembled. Combining the results of both approaches, 65,561 transcripts were identified for CHO cell lines, which could be clustered by sequence identity into 17,598 gene clusters.

## Background

The Chinese hamster, *Cricetulus griseus*, was introduced as a laboratory animal in 1919 [1]. In 1957 it was the donor of the first Chinese hamster ovary (CHO) cell line [2]. Nowadays, related CHO cells are the most commonly used cell lines in modern research and biotechnology [1]. As mammalian expression systems, they are widely used for the industrial production of therapeutic proteins, because they perform complex folding and post-translational modifications of proteins that are not immunogenic in humans [3]. The application of CHO cells in the large-scale production of pharmaceutical proteins generates revenues of billions of dollars each year with numbers constantly rising [4]. Due to the increased usage of CHO cells, knowledge about the transcriptome of the cell lines is an important need. Little information on the transcriptome sequence of CHO cells was available in public databases until recently. Consequently, transcriptome analyses for example by applying DNA microarrays were not available to a broad scientific community, despite the importance of CHO cells for research and biotechnology. Therefore, previous attempts to analyze the transcriptome of CHO cells, relied on cross species hybridizations with microarrays designed for the closely related species mouse or rat, putting up with disadvantages such as decreased sensitivities [5] [6] [7]. To overcome these limitations, genome and transcriptome sequencing is a valuable tool in modern research and biotechnology. While sequencing projects have mainly been Sanger-based in the past, next-generation sequencing (NGS) technologies represent a time and cost efficient alternative today [8].

In 2009, a combined sequencing approach was applied to generate 68,000 Sanger-based expressed sequence tags (EST) and 400,000 Roche/454 NGS reads to assemble ∼28,000 unique CHO cell sequences [9]. These sequences were used to establish a custom CHO cell Affymetrix array for transcriptome analysis [9] and as a reference assembly for RNA-seq-based gene expression profiling [10], but the sequencing results and the array itself are not available to the public. One year later, Birzele and colleagues used Illumina’s sequencing approach to prove that large-scale expression profiling for CHO cells is possible using NGS technologies. 13,375 genes were identified in this study, but only short read data was deposited to the NCBI Short Read Archive [11]. In 2011, Illumina’s NGS was used to sequence the first two CHO cell line genomes in two independent studies. The CHO-SEAP genome was sequenced with one-fold coverage, only [12]. Assembly of the data therefore was performed with help of publicly available reference genomes of mouse and rat. Due to the low sequencing coverage, assembly of the data yielded a relatively high number of 3.57 million sequence contigs. Nevertheless, 17,883 homologs of mouse genes and 19,500 homologs of rat genes were identified by this approach. From this study, short reads were published [12]. In a second study, CHO-K1 was sequenced with a coverage of 100-fold [13]. *De novo* assembly of the data generated 109,151 scaffolds and 265,786 contigs. The genome size of CHO-K1 was estimated at 2.45 Gb and 24,383 genes were predicted from the draft genome with the help of 10.8 Gb of transcriptome sequencing data [13]. With this study, assembled genome data of CHO cells was made publicly available for the first time. Shortly after, Becker and coworkers [14] deposited the first assembled transcriptome data from CHO cells in the NCBI database. In this study, 1.84 mio reads were sequenced with Roche’s NGS approach and assembled with the GS *De Novo* Assembler version 2.5. This assembler addresses the characteristic needs of eukaryotic transcripts, like exon and intron structures and alternative splice sites. This approach generated 29,184 possible transcripts and 24,576 possible genes. Taxonomic classification showed that more than 70% of this data is homologous to the transcriptome of mouse and that metabolic pathways like the central carbohydrate metabolism are almost completely represented by the transcriptome data [14]. Due to the progress in sequencing technologies and assembly algorithms, new studies focused on the establishment of draft genomes from Chinese Hamster or CHO cell lines [15] [16]. Despite the recent rise in publicly available sequence information, proper assembly and annotation of these data sets is still a work in progress.

The present study aims at developing an improved transcript data set for CHO cells, based on available transcriptome data [14] and additional sequencing data generated using Roche’s and Illumina’s NGS approaches. Hybrid assemblies of different data sets are challenging due to the variable read lengths, the dissimilar sequence coverage, and the different sequencing errors of the NGS approaches used [17]. In contrast, a reference-based assembly using the published CHO-K1 genome can help to assemble full-length transcripts. Since the genomic sequence is split in many scaffolds containing gaps, however, some transcripts will not be assembled completely or will be missed. To address these challenges, we developed a two-branched assembly pipeline combining *de novo* and reference-based assemblies into one final transcriptome set for CHO cells. This approach is complemented by the public available web-based annotation systems, GenDBE and SAMS, for browsing genomic and transcriptomic data, respectively, thus increasing the usability of the information for the scientific community.

## Results and Discussion

### Illumina and Roche/454 RNA Sequencing

Becker et al. published a first transcript data set from Chinese hamster ovary (CHO) cell lines in 2011 [14]. In order to extend and improve this transcript set, NGS technologies from Roche/454 and Illumina were applied to sequence normalized cDNA libraries constructed from CHO-K1 mRNA samples. CHO-K1 cells were cultured in four independent fermenters, one exposed to temperature stress and one exposed to pH-shift to include a broad range of diverse transcripts. Samples were taken throughout the growth curve and pooled prior to mRNA isolation and sequencing library construction.

A total of 1,249,862 reads were sequenced using Roche’s Genome Sequencer FLX with Titanium chemistry. Additionally, 47,235,395 reads were sequenced with Illumina’s Genome Analyzer IIx applying 2×150 bp paired end sequencing mode. After trimming low quality ends a mean length of 333 bp for the Roche/454 reads and 106 bp for the Illumina reads remained for the following assembly steps. These sequencing data were complemented with 1,837,072 Roche/454 reads from the previous work from Becker and coworkers (Table 1).

**Table 1:** Next-generation RNA sequencing data from CHO cell lines analyzed.

CHO cell line	Sequencing method	Number of reads	Mean read length [bp]	Reference
CHO-K1/DUKXB11	Roche/454 GS FLX, Titanium	1,837,072	328	[14]
CHO-K1	Roche/454 GS FLX, Titanium	1,249,862	343	this work
CHO-K1	Illumina GAIIx, 2×150 bp(paired end)	47,235,395	82/130	this work

### Two-tiered Assembly Pipeline

**Figure 1:**
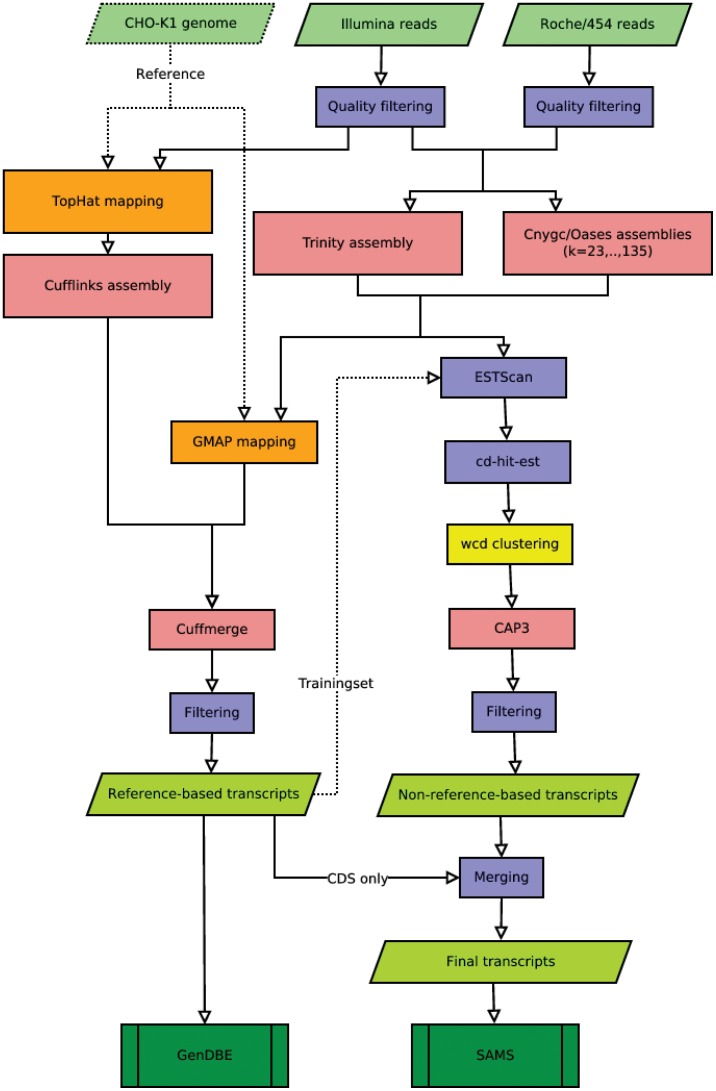
Workflow for the reference-based and the non-reference-based re-assembly of CHO cell transcripts. The left side shows the reference-based pipeline, the right side the non-reference-based pipeline. Different colors represent the different processes: assembly steps, red; mapping steps, orange; filtering steps, light blue; clustering, yellow; raw data, light green; annotated transcriptome data, dark green. Final transcriptome data is publicly available via the GenDBE [26] and SAMS [27] web tools.

Recent studies have shown that a combination of *de novo* and reference-based strategies yields the best outcome for transcriptome assemblies [18] [19] [20]. Accordingly, we developed a two-tiered pipeline consisting of reference-based and non-reference-based methods to create our transcript database. A detailed overview of the pipeline is shown in Figure 1.

### *De novo* and Reference-based Transcriptome Assembly

**Figure 2:**
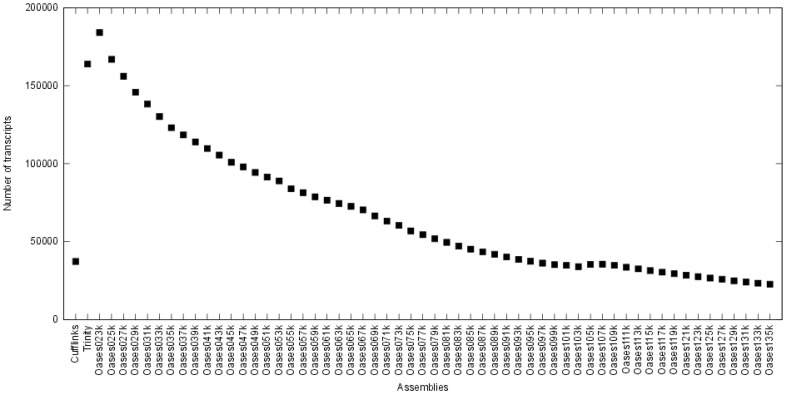
Number of CHO cell transcripts assembled with Cufflinks, Trinity, and Oases. K-mer sizes vary between 23 and 135 for the Oases assembly.

**Figure 3:**
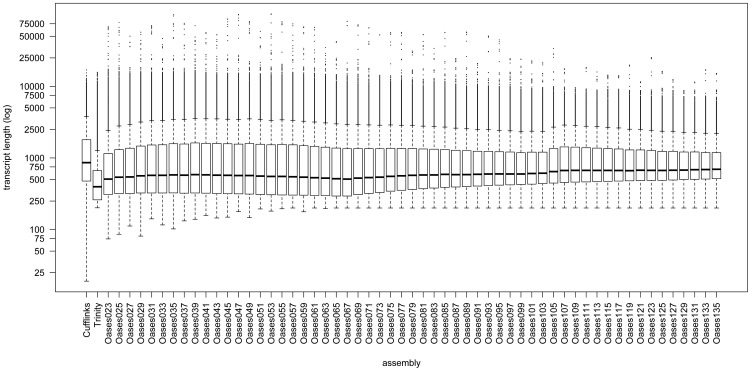
Length distribution of the transcripts assembled with Cufflinks, Trinity, and Oases.

In general, the method and options used to assemble sequence reads, like the k-mer value chosen for de Bruijn graph based *de novo* assemblers, have a strong influence on the result of an assembly. To take advantage of different assembly tools, we used Cufflinks, Trinity, and Oases with multiple k-mer sizes to assemble all sequencing data accessible for CHO cells. By this means, 59 individual assemblies were carried out. Figure 2 shows the number of assembled transcripts by each method and Figure 3 depicts the length distribution of the transcripts, respectively. The total number of transcripts assembled by the reference-based assembler Cufflinks is 37,363, which is significantly less than the mean number of transcripts calculated from the 57 *de novo* assemblies computed with Oasis (mean: 68,520.34, maximum: 184,030, minimum: 22,792). Thus, the mean length of the transcripts is higher in the reference-based Cufflinks assembly (reference-based assembler: 1,353 bp, *de novo* assembler: 1,151 bp). Results of the Trinity assembler showed the shortest transcripts with a mean length of 767 bp.

**Figure 4:**
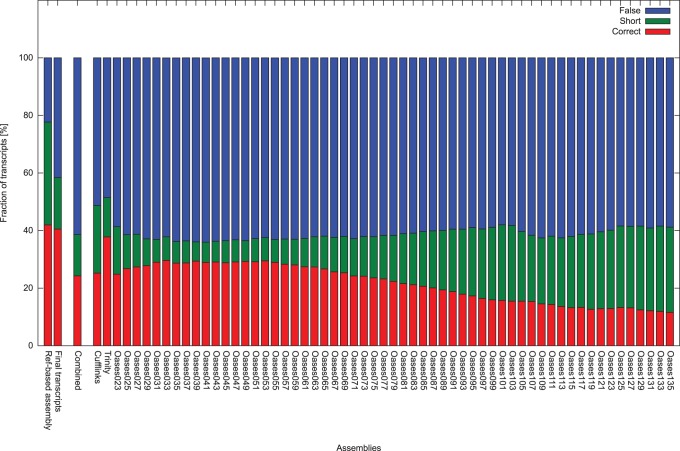
Comparison of the proportions of correctly assembled transcripts and misassemblies. All transcripts with significant BLASTp hit against the mouse reference protein set were classified into “correct” (red), “short” (green) and “false” (blue) assembled transcripts.

*De novo* transcriptome assemblers produce a large number of misassembled or incomplete transcripts [21]. To estimate the proportion of correctly assembled transcripts, a BLAST search against a set of non-redundant mouse proteins was performed. All transcripts with a significant best hit (e-value ≤10^−20^, percent identity ≥90%) were checked. Hits covering more than 90% of the reference protein with less than 1% gaps were marked as correct, hits covering the reference protein by less than 90% but covering more than 90% (gaps ≤1%) of the transcript were marked as incomplete, and all other hits were marked as false assemblies. The Trinity Assembler performed best in this respect with 38% correct and 14% incomplete transcripts (Figure 4). The result of Cufflinks (25% correct, 24% incomplete) is comparable to the best Oases results (ranging from 30% to 12% correct, and 30% to 7% incomplete).

**Figure 5:**
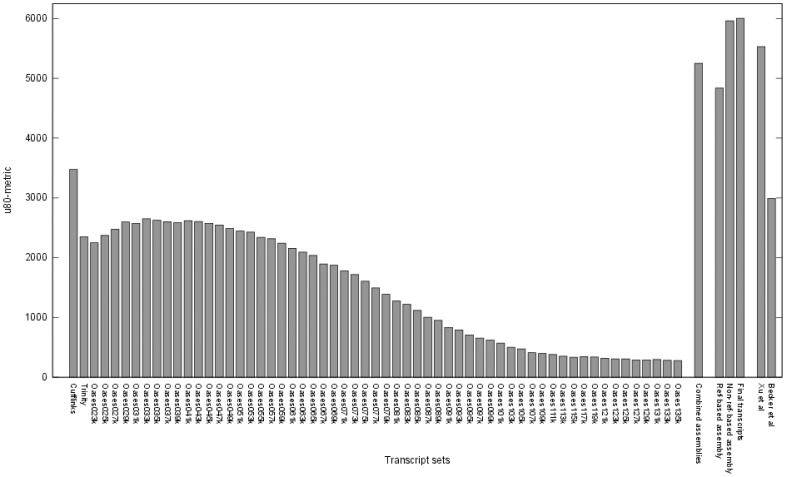
“u80-metric” comparison of individual transcriptom assemblies. The comparative u80-metric results for the single Cufflinks, Trinity and Oases assemblies, the combined assemblies, the results of the reference-based re-assembly (ref-based), the non-reference-based re-assembly (non-ref-based) and the final transcript set (final transcripts) are compared to two publicly available CHO cell transcript sets, Xu et al. [13] and Becker et al. [14].

**Figure 6:**
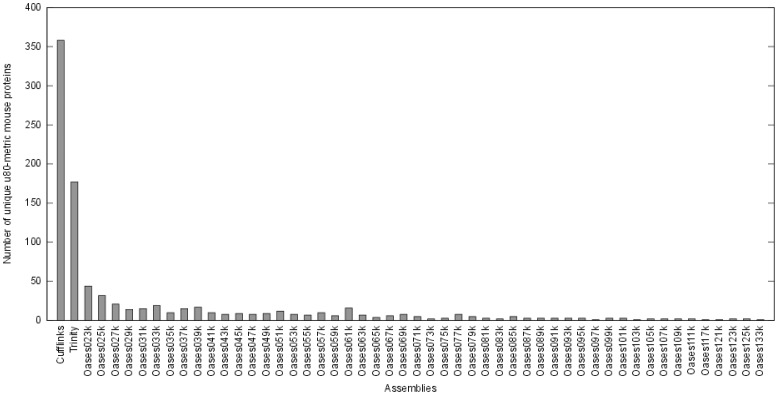
Unique u80-metric mouse proteins for the individual assemblies. Almost all individual assemblies (52 of 59) have transcripts with an ungapped alignment covering a mouse protein by more than 80% that are not present in the other assemblies.

To compare the different assemblies, a new metric (u80-metric) was introduced. The set of non-redundant mouse reference proteins was aligned to the transcripts assembled with the different assembly tools. For each assembly, the number of reference proteins with an ungapped alignment covering at least 80% of the protein was counted. As this metric is only used to compare the different assemblies in terms of correctly assembled transcripts, the parameter were chosen quite stringent to reduce the number of false positives. The resulting values range from 2,625 to 278 for the different *de novo* assemblies of Trinity and Oases. Figure 5 gives an overview of the u80-metric values of all assemblies and the combined data sets. Cufflinks shows the best performance with respect to the u80-metric with a value of 3,474. However, the u80-metric value of the combined assemblies, which was computed on the set of all sequences of the assemblies is about 50% higher than the best single assembly, with a value of 5,252. In contrast the proportion of correctly assembled transcripts (24% correct, 14% incomplete) is lower than in the Trinity (38% correct, 14% incomplete), Cufflinks (25% correct, 24% incomplete), and the best Oases assemblies (30% correct, 8% incomplete) (Figure 4). These numbers lead to the conclusion that no single assembly approach, even based on a reference draft genome, is sufficient to cover the complete transcriptome. This is supported by the number of unique u80-metric mouse proteins (mouse proteins that fall into the u80-metric in exactly one assembly set). As shown in Figure 6, 52 of the 59 assemblies have at least one unique match to a mouse protein and only some Oases assemblies with k-mer values larger than 111 do not contain any unique sequences falling into the u80-metric. Therefore the combined assemblies, with about 4 million sequences were used for the following steps.

### Reference-based Re-assembly Strategy

A reference-based approach to re-assemble the complete data set was developed in order to filter out redundant sequences and assembly errors. With this approach all transcripts from the *de novo* assemblies computed by Trinity and Oases were mapped to the draft genome sequence of the CHO-K1 cell line [13] and re-assembled using the cuffmerge tool [22]. A total number of 28,874 transcripts located on 16,412 unique gene loci were predicted for this reference-based re-assembly. The transcripts have a mean length of 3,098 bp and a mean CDS length of 1,487 bp (predicted using the “longest ORF” approach). About 16% (4,823) of the transcripts are single-exon transcripts. A mean number of 12 exons per multi-exon transcript and a maximum of 118 exons per transcript were observed.

The respective u80-metric value of the reference-based re-assembly of 4,836 shows an improvement in comparison to the single assemblies summarized in Figure 5, but still is smaller than the u80-metric of the combined assemblies. This may be due to the draft state of the CHO cell genomic reference sequence or errors during the mapping.

### Non-reference-based Re-assembly Strategy

Additionally to the reference-based re-assembly a non-reference-based approach was developed to address the draft status of the CHO-K1 reference genome sequence. After CDS prediction on the sequences of the Cufflinks, Trinity, and Oases assemblies, removal of redundant sequences and re-assembly, the number of sequences could be reduced from over four million to 142,098 with an u80-metric value of 5,959 (Figure 5). Similar to the individual assemblies, the non-reference (*de novo*) method produces a larger number of sequences than the reference-based strategy, since minor sequence differences like insertion or deletions based on sequencing errors cannot be resolved by these methods.

### Generation of the Final Transcript Data Set

By removing transcripts of the non-reference-based re-assembly, which are completely covered by a gapped alignment of transcripts from the reference-based re-assembly, the total number of transcripts was reduced to 65,561 in the final transcript data set. These transcripts were clustered into 17,598 clusters using wcd [23]. On the one hand the clustering approach was used to group isoforms of a gene, but on the other hand it might have also clustered paralogous genes or gene families. Therefore, a cluster can represent multiple gene loci. The number of multiple gene loci in a single cluster was estimated by mapping the transcripts back to the reference genome and counting of gene loci for each cluster. Most clusters (14,534 clusters, 83%) could be mapped to the same gene locus (Table 2), 1,818 clusters (10%) were mapped to multiple gene loci, likely containing paralogous genes, and 971 clusters (5%) could not be mapped to the genome. The mean length of 1,665 bp for transcripts of the final data set is smaller than the mean length of transcripts in the reference-based re-assembly, because the respective pipeline produces CDS regions only. This approach increases the u80-metric value to 6,003 for the final transcript data set, which is even higher than the u80-metric value for the combined assemblies. Although combining different assembly methods increases the number of correctly assembled transcripts, it could be shown that some biological information might be lost [24]. To test this effect, the set of reference mouse proteins was blasted against the translated ORFs of all assemblies and against the protein sequences of the final transcript set. A reference protein was considered to be present in the combined assembly set, if a BLAST hit was found with an e-value ≤10^−5^. For the final data set, a protein was counted as present, if a BLAST hit was found with an e-value ≤10^−5^ and the percent identity of this hit was not more than 5 points below the percent identity of the BLAST hit against the combined assembly set. With this method 30,651 reference proteins were identified within the combined assembly set, 1,860 (6%) of these were missing in the final set.

**Table 2:** Estimation of the number of cluster with paralogous genes.

	Single genelocus	Multiplegene loci	No gene locus
**Unique cluster**	14,415	1,698	971
**Multiple cluster**	119	120	–

If transcripts from different cluster mapped on the same gene locus, the transcripts where counted as “multiple cluster”.

### Comparison of the Transcript Data Sets to Existing CHO Cell Transcriptome Data

**Figure 7:**
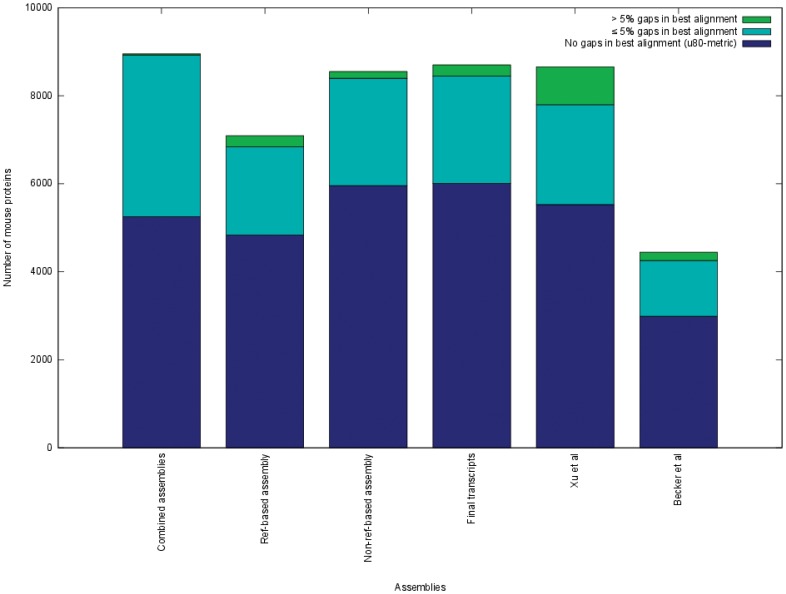
Comparison of the different transcript sets. The comparative metrics values of the combined assemblies, the results of the reference-based and non-reference-based pipelines and the final transcriptome set. For comparison the results of published CHO transcriptome sets are also shown (Xu et al proteins [13] and Becker et al *de novo* assembly [14]).

For comparative analysis, the u80-metric was also computed on the protein sequences published along with the CHO-K1 draft genome [13], resulting in a value of 5,528 (Figure 5). Since the respective protein set was produced using a *de novo* gene prediction strategy, in addition to transcriptomic data, genes that are expressed in the Chinese hamster but not in the CHO cell lines analyzed, might also be part of the data set. The data set by Xu et al. does not contain different isoforms of genes. Because of these two major differences a direct comparison of the u80-metrics may not be meaningful, whereas, extending the metric by allowing gapped alignments shows a significantly higher number of mouse reference proteins with larger (>5%) gaps in the alignment. This could be an indication that the *de novo* gene prediction is missing some exons (Figure 7, green bars).

**Figure 8:**
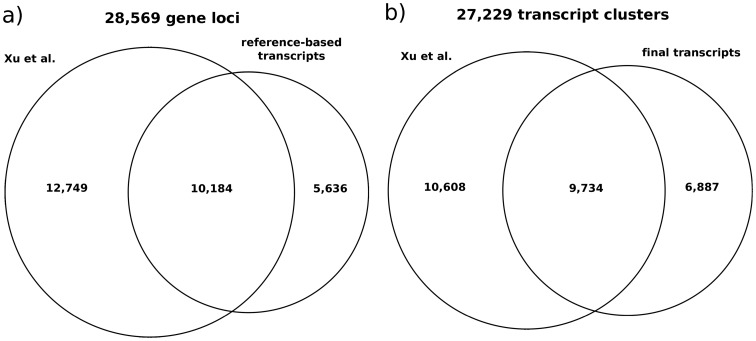
Comparison of the public transcript set from Xu et(a) the reference-based re-assembly and (b) the non-reference-based re-assembly. The Venn diagram (a) gives an overview of the predicted unique gene loci of both data sets. More than 1/3 of the transcripts are present in both sets. The venn diagram (b) shows the gene clusters created with the wcd [23] tool.

Our reference-based pipeline predicted 5,636 new putative gene loci in addition to the 24,238 gene loci predicted by Xu et al. (Figure 8a), extending the total set of putative gene loci in the CHO-K1 genome to 28,596. A total of 10,184 gene loci could be identified in both datasets. In some cases a transcript could be mapped to more than one unique Xu et al. gene locus merging them to a single locus. Thus 2,468 Xu et al. gene loci could be merged to 1,163 gene loci. A clustering of the Xu et al. transcripts and our final transcript set using wcd produced 27,229 clusters (Figure 8b). About 35% (9,734) of the clusters contain at least one transcript from both data sets. 6,887 new clusters could be introduced by our non-reference-based approach. Transcripts of 6,106 of these clusters could be located on the reference genome with only 191 overlapping with Xu et al. gene loci.

### Automatic Functional Annotation

**Figure 9:**
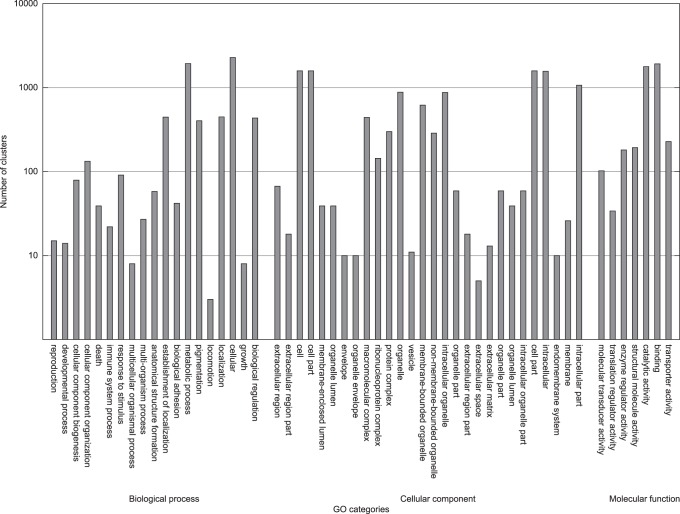
GO category distribution of the gene clusters from the final transcript set using all annotated GO terms up to the second level.

An automated annotation pipeline involving BLAST and HMM searches to different databases was used for the annotation of the final transcript data set. Functional annotations were assigned for 51,045 of the transcripts (77%), which correspond to 10,643 of the 17,598 clusters (60%). For the 5,636 new gene loci 6,302 transcripts were predicted, of which 2,954 (52%) could be functionally annotated. Functional annotation of the 10,953 transcripts in the new clusters revealed 3,044 annotated transcripts (28%).An overview of the assigned GO categories is shown in Figure 9. Additionally, possible transposable elements were searched, using RepeatMasker. A total of 3,177 transposable elements were detected (2,040 LINE, 353 SINE and 831 LTR), which were clustered into 495 clusters, containing 3,979 transcripts.

### Public Web-interfaces

**Figure 10:**
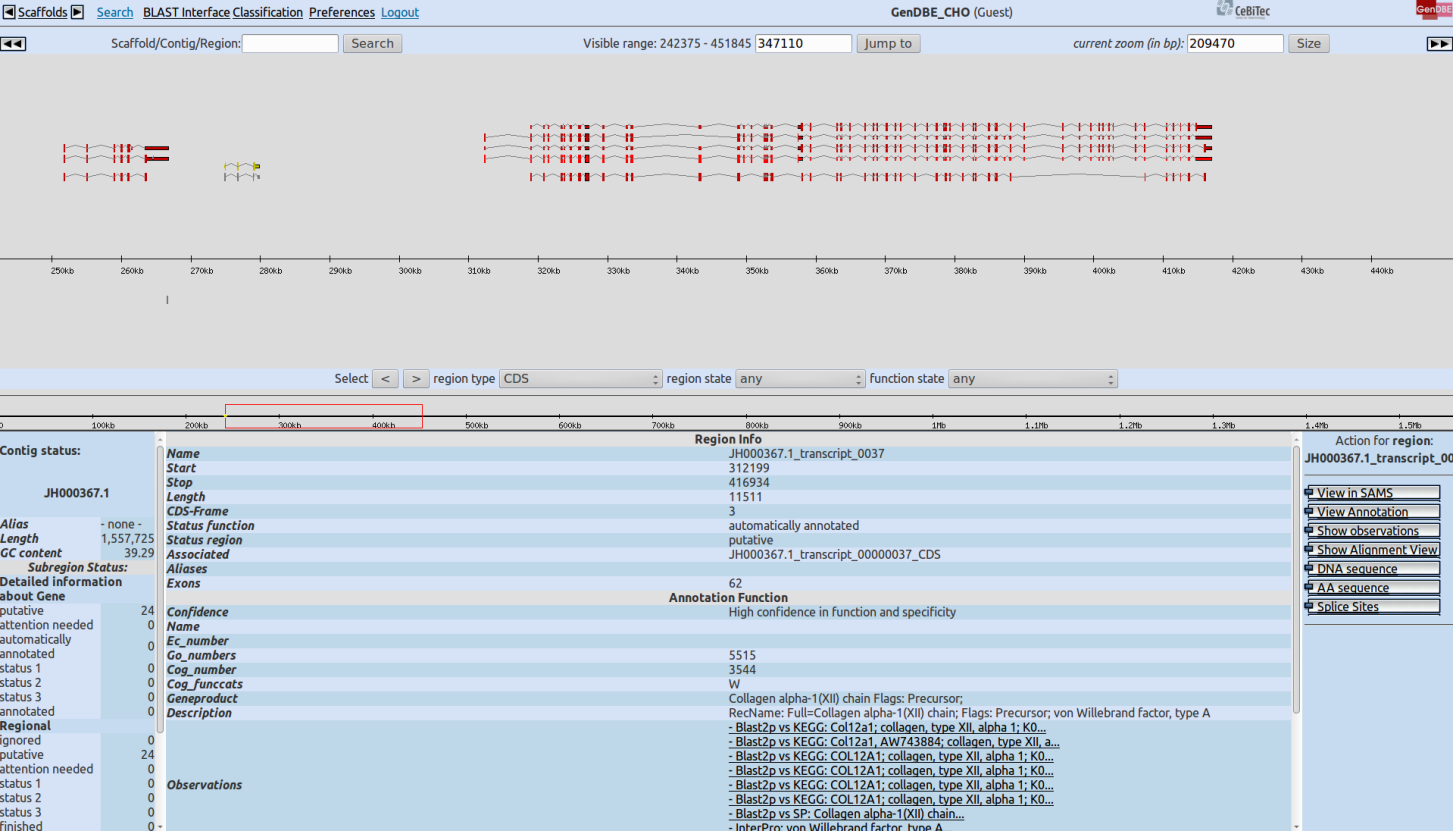
Screenshot of the GenDBE web-interface. The upper main area of the web-interface shows a graphical representation of a genomic contig with the exon/intron structure of the annotated genes. Informations of a selected gene or transcript are shown in the lower part in the center. All available actions, that can be performed on the selected gene, are listed on the bottom right part of the main window. The menu on the top of the interface gives the user different means to browse the complete database.

**Figure 11:**
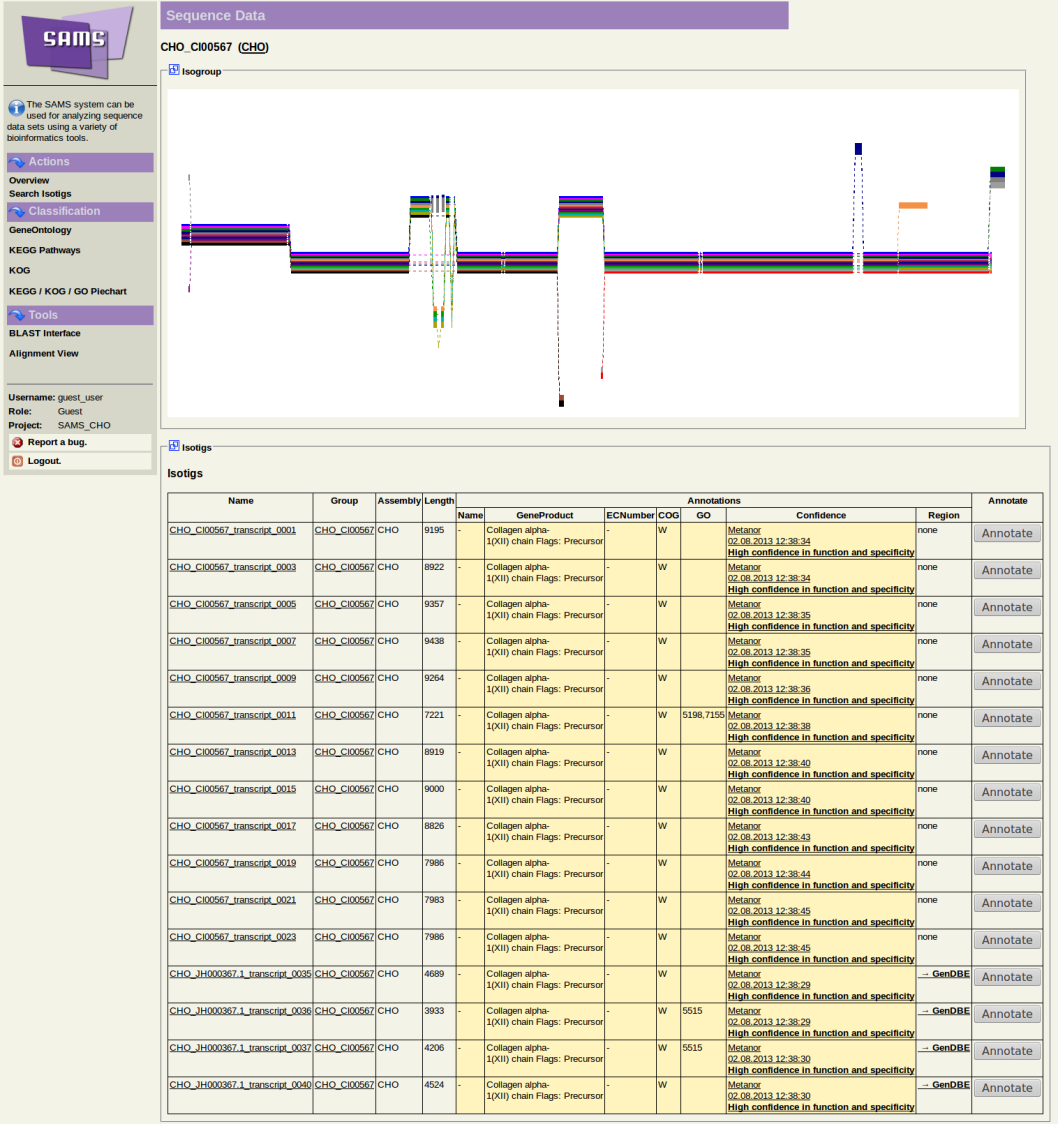
Screenshot of the SAMS web-interface showing a single transcript cluster. The upper half of the interface shows a graphical representation of a possible splicing-graph of the cluster. The lower part lists all transcripts of the cluster with some information about the functional annotation of the transcript. Links to browse the database are on the left side of the interface.

Two web-based interfaces were established to allow users to browse the complete transcriptome data set by different means. The prokaryotic annotation system GenDB [25] was extended for eukaryotic genomes. A graphical representation of the reference-based transcripts and their intron-exon structures on the CHO-K1 draft genome is publicly available at the GenDBE web-interface [26] (https://gendbe.cebitec.uni-bielefeld.de/cho.html) (Figure 10). Additionally, the final transcripts can be browsed using the SAMS web-interface [27] (https://sams.cebitec.uni-bielefeld.de/cho.html) (Figure 11), which is an extended version of the original SAMS system [28]. A visualization of a possible splicing-graph, representing the splice variants of the cluster, computed from the multiple alignment of the amino-acid sequences using POA [29] and POAVIZ [30], is available for each cluster. Both interfaces provide comprehensive search functionality. Annotated EC numbers allow a mapping of the data onto KEGG pathway maps. The transcripts can be filtered by GO or KOG terms. A BLAST interface with DNA and amino-acid databases of the transcripts is also provided by both interfaces.

## Conclusion

With this study we introduce two web-based tools, GenDBE and SAMS, to browse and analyze the high quality CHO cell transcript database that was constructed using a two-tiered pipeline for the assembly of RNA-seq reads with and without the use of a reference genome sequence. By applying this pipeline to CHO cell RNA-seq data from different sequencing technologies, we could improve and extend the publicly available CHO cell transcriptome. It was shown that a single assembly, even guided by a draft genome, is not sufficient to construct a complete set of transcripts. In general, the *de novo* assembly methods produce a higher number of transcripts than the reference-based methods. A possible reason for this is that the *de novo* assemblers will not assemble reads from transcripts of a heterozygous gene to a single sequence. In contrast, the reference CHO-K1 genome is haploid so that allelic reads will map to the same genomic locus. Therefore, a reference-based assembler produces only a single sequence for each splice variant. Furthermore, the reference-based assembled sequences might be incomplete due to gaps in the reference sequence. It has also been shown that the k-mer value has a great influence on the results of the *de novo* assemblers. In our analysis, almost every individual Oasis assembly contained unique sequences with a high homology to a reference mouse protein without any gaps in the alignment and thus could be considered as correctly assembled. Taking advantage of the different abilities of the assembler tools used and merging the results of all the different assemblies, a CHO cell transcriptome data set as complete as possible has been created. For practical reasons not all of the available tools and methods to assemble transcript sequences from RNA-seq data were used in this study. Known mouse or rat transcript sequences for example, as introduced by Birzele et. al [11], might be used as a reference to assemble the CHO homologs to these transcripts. The developer team of the Trinity assembler just released a first beta version of a “genome-guided Trinity” which uses a combination of read mapping to a reference genome and *de novo* assembly of the reads that map to the same partition of the genome. This approach combines the advantages of reference-based and *de novo* assemblies within one tool. New studies to further improve the data might follow. However, new data sets can be easily incorporated into our database to establish an up-to-date analysis platform, e.g. the protein data set published with the CHO-K1 genome is also available in the GenDBE database.

## Methods

### CHO Cell Line and Culture Conditions

A serum-free adapted sub clone of the CHO-K1 parental cell line (ATCC CCL-61) was cultured in two 2 l glass fermenters with pH and pO_2_ adjustment control in a starting volume of 800 ml in TC-42 medium (Teutocell AG, Bielefeld, Germany) supplemented with 6 mM L-glutamine and 1×HT supplement. Feeding started 48 h after initiation of the fed-batch process at 37°C, pH 7.05, and 40% humidity. Two experimental setups of cultivation were done, where cells were exposed to either temperature stress or pH-shift to obtain a broad range of diverse transcripts. Simultaneously, a reference cultivation under standard conditions was conducted for both experiments. In setup one, the initial pH was shifted to 6.9 after 72 h of cultivation by the addition of NaHCO_3_. In setup two, temperature was shifted to 33°C after 72 h. From this time point on, samples of 1×10^7^ cells were harvested each day by centrifugation at 600×g. Samples were stored at −80°C.

### cDNA Library Construction and Next-generation Sequencing

RNA purification and cDNA libraries were prepared by Vertis Biotechnology AG (Freising, Germany). Normalized libraries for both Roche/454 and Illumina sequencing were synthesized from poly(A)^+^ RNA using random primers to equally cover the transcripts. The quantities of the cDNA libraries were evaluated with the Quant-iT PicoGreen dsDNA kit (Invitrogen, Carlsbad, USA) and the Microplate Reader Tecan Infinite 200 (Tecan Trading AG, Männedorf, Switzerland).

For the Roche/454 cDNA library, fragment sizes range from 550–800 bp. The DNA library was amplified by emulsion PCR and sequenced on the GS FLX system using Titanium sequencing chemistry according to the manufacturer’s instructions over one sequencing plate (Roche Applied Sciences, Mannheim, Germany).

For Illumina paired end sequencing (2×150 bp), fragment sizes range from 400–500 bp. The sequencing was performed using the “Genome Analyzer IIX” (GAIIX, Illumina Inc., San Diego, CA, USA) by IIT Biotech GmbH (Bielefeld, Germany).

The Roche/454 reads were trimmed and converted into FASTQ file format using the sffinfo tool (Roche Applied Sciences). Illumina reads were trimmed using the FASTX-toolkit [31] with a minimum phred score of 15.

### Transcriptome Assembly Strategies

Different assembly methods were applied to construct transcript sequences from the reads. For a reference-based approach the Illumina reads were mapped to the CHO-K1 draft genome published by Xu and coworkers [13] with TopHat (version 1.4.1, default parameters) [32]. The resulting mapping was assembled with Cufflinks (version 1.2.0, default parameters) [22].

Additionally, two different *de novo* assembly methods were used. One assembly was computed with the Trinity transcriptome assembler (release 2011-10-29) [33] combining Illumina and Roche/454 reads, including all data as single end reads. For the second *de novo* assembly, the Oases assembler (version 0.2.01) [34] was used with the Convey (Convey Computer Corporation) FPGA (Field Programmable Gate Array) implementation of the Velvet algorithm (cnygc version 1.1) using all odd k-mer values between 23 and 135 (Illumina reads were marked “short paired end” and Roche/454 reads “long”), resulting in 57 individual assemblies.

### Reference-based Re-assembly

Re-assembly of the individual assemblies was carried out to merge the different assemblies. This was achieved with a reference-based pipeline and a non-reference-based pipeline. For the reference-based pipeline, the *de novo* assemblies with Trinity and Oases were first mapped to the reference CHO-K1 [13] genome using GMAP (release 2011-12-13, default parameters) [35]. Transcripts with less than 90% mapping coverage were removed. The resulting 59 mappings (Cufflinks, Trinity, and 57×Oases) were merged to a single mapping with the cuffmerge tool provided by the Cufflinks distribution. Transcripts with false (non-canonical) splice sites and transcripts with their longest ORF less than 75% of the length of the longest ORF in the gene locus were removed.

### Non-reference-based Re-assembly

In addition to the reference-based approach a non-reference-based pipeline was used to merge the initial Cufflinks, Trinity, and Oases assemblies. First, the complexity of the assembled sequences (forward/reverse sense sequences, UTR, CDS, and intron sequences) was reduced by CDS detection using ESTScan version 3.0.3 [36]. A training set of 1,802 transcripts for ESTScan was derived from a random selection of the transcripts with the longest ORF per gene locus created by the reference-based pipeline. ESTScan then was applied on all transcripts with default parameters. In the second step, the ESTScan results were screened for redundant sequences with cd-hit-est version 4.5.6 [37]. Transcripts with an ungapped alignment covering at least 90% of the complete length were removed from the set since they are included in a longer sequence. The remaining transcripts were clustered using wcd [23] with default parameters.

A re-assembly of the transcripts from the individual assemblies was performed in the third step using CAP3 [38]. The assembly was performed for each cluster separately with options for forward alignments only (-r 0) to prevent chimeric assemblies and a large gap penalty factor (-n 10000) to prevent the introduction of new frame shifts.

### Merging the Pipeline Results to the Final Transcript Data Set

The redundant transcripts of both pipeline results were removed by aligning the reference-based transcripts to the non-reference-based transcripts. Non-reference-based transcripts were removed, if an alignment with coverage of at least 95% was found. Thereby, all transcripts with minor differences to the reference genome were removed. A reference-based transcript was removed only, if it was completely covered by a non-reference-based transcript without gaps.

### Estimating the False to Correct Assembly Ratio

A BLAST search against a set of non-redundant mouse proteins downloaded from Ensembl (version 68) was performed to estimate the proportion of correctly assembled transcripts in each data set. The *de novo* assemblies were screened for all possible open reading frames (ORF) ≥200 nt. The ORFs were translated into protein sequences and aligned to the mouse reference set using BLASTp. The same analysis was performed on the reference-based and the final data set, but here only the predicted coding sequences were translated to the protein sequence and aligned. The best significant hit (e-value ≤10^−20^, percent identity ≥90%), was used to classify the transcript. If more than one ORF of a transcript had a significant hit, the hit with the highest score was chosen. The transcript was marked as correctly assembled, if the hit covered the reference protein ≥90% and with ≤1% gaps. Hits with ≤1% gaps covering the reference <90% but covering the transcript ≥90% were marked as incomplete assemblies. All other transcripts with a significant hit were marked as misassemblies. The fraction of correctly assembled transcripts was computed as the number of transcripts marked as correct divided by the number of transcripts with a significant hit.

### Construction of the Reference Metric

A simple metric was used to compare the different assemblies. To compute this metric, the set of non-redundant mouse proteins was aligned to the transcripts using blat [39] with parameters “-q = prot -t = dnax -minIdentity = 70” for the *de novo* assemblies and “-prot -minIdentity = 70” for the translated amino acid sequences of the transcripts derived from the two pipelines. Alignments covering at least 80% of the reference protein were classified by the percentage of gaps in respect to the alignment length (0%, ≤5%, and >5%) for each reference protein. We defined the u80-metric as the number of reference proteins with ungapped alignments with at least 80% coverage.

### Estimating the Number of Paralogous Genes in a Cluster

To estimate the number of paralogous genes that fall into the same cluster, all transcripts were mapped to the reference genome using gmap [35] with the parameter “-n 1” to get the best gene locus for each gene. The resulting gene loci in GFF file format were clustered using the gffread tool from the Cufflinks [22] distribution with the “–cluster-only” parameter. The number of unique gene loci were counted for each cluster and vice versa. The number of transcripts from different cluster, that mapped to the same unique gene locus, were also counted.

### Extending the SAMS and GenDBE System

SAMS and GenDBE are based on the same extended GenDB-backend [25]. GenDB uses a mysql database with an object-oriented API (O2DBIv2 [25]), where all sequences are stored in the Region::Source class. Each sequence feature (e.g. genes) are subregions of a Region::Source object.

Three new classes were introduced for the SAMS system: Region::Isogroup, Region::Group::Isotig and Region::Source::NGSContig, based on the nomenclature of the GS *de novo* Assembler (Roche Applied Sciences). The NGSContig object stores the sequence information that is shared between different splice variants. The Isotig object represents a specific splice variant and, in contrast to the original GenDB-backend, the sequence information is derived by the concatenation of NGSContig objects. The Isogroup object is used to group all splice variants of a gene or all transcripts of a cluster.

The GenDBE system uses the Region::Group::Isotig class to represent spliced features. Each exon is represented in a “traditional” GenDB feature (subregion of the Region::Source object) and the sequence of the spliced feature is derived by concatenation of the exon sequences.

### Clustering and Functional Annotation

The reference-based transcripts were uploaded to GenDBE. The non-reference-based transcripts were clustered using the wcd clustering tool [40]. The clusters were then uploaded to the SAMS system [28]. The automatic annotation pipeline Metanor-Euk [41] was applied on both data sets with different BLAST [42] searches against various databases, including SwissProt [43], KEGG [44], KOG [45], and eggNOG [46], and HMM based tools such as Pfam [47], InterProScan [48], and Panther [49] for functional prediction. The pipeline chooses the best result from each tool (e.g. based on e-value and score for BLAST results, cutoff 10^−10^) and assigns a confidence value based on the quality of the result. The best results of all tools are combined and the functional annotation (gene name, EC number, GO number, etc.) is extracted, where results with better confidence values are preferred. Additionally the transcripts were screened for possible transposable elements with RepeatMasker (version 4.0.3).

### Data Access

The RNA-seq data sequenced in this study have been deposited at the European Nucleotide Archive under accession number PRJEB4847 (http://www.ebi.ac.uk/ena/data/view/PRJEB4847). The transcripts and the functional annotation can be browsed and downloaded in different formats at the GenDBE (https://gendbe.cebitec.uni-bielefeld.de/cho.html) and SAMS (https://sams.cebitec.uni-bielefeld.de/cho.html) project pages.
